# Emotion Recognition With Knowledge Graph Based on Electrodermal Activity

**DOI:** 10.3389/fnins.2022.911767

**Published:** 2022-06-09

**Authors:** Hayford Perry Fordson, Xiaofen Xing, Kailing Guo, Xiangmin Xu

**Affiliations:** ^1^School of Electronic and Information Engineering, South China University of Technology, Guangzhou, China; ^2^School of Future Technology and the School of Electronic and Information Engineering, South China University of Technology, Guangzhou, China

**Keywords:** affective computing, electrodermal activity, knowledge graph, emotion recognition, MLP

## Abstract

Electrodermal activity (EDA) sensor is emerging non-invasive equipment in affect detection research, which is used to measure electrical activities of the skin. Knowledge graphs are an effective way to learn representation from data. However, few studies analyzed the effect of knowledge-related graph features with physiological signals when subjects are in non-similar mental states. In this paper, we propose a model using deep learning techniques to classify the emotional responses of individuals acquired from physiological datasets. We aim to improve the execution of emotion recognition based on EDA signals. The proposed framework is based on observed gender and age information as embedding feature vectors. We also extract time and frequency EDA features in line with cognitive studies. We then introduce a sophisticated weighted feature fusion method that combines knowledge embedding feature vectors and statistical feature (SF) vectors for emotional state classification. We finally utilize deep neural networks to optimize our approach. Results obtained indicated that the correct combination of Gender-Age Relation Graph (GARG) and SF vectors improve the performance of the valence-arousal emotion recognition system by 4 and 5% on PAFEW and 3 and 2% on DEAP datasets.

## 1. Introduction

Emotions are vital for humans because they influence affective and cognitive processes (Sreeshakthy and Preethi, 2016). Emotion recognition (Li et al., 2021) over the years has received large attention from academic researchers and industrial organizations and has been applied in numerous sectors including transportation (De Nadai et al., 2016), mental health (Guo et al., 2013), robotics (Tsiourti et al., 2019), and person identification (Wilaiprasitporn et al., 2020). Emotions are accompanied by physical and psychological reactions when an external or internal input is introduced. Representative methods for emotion recognition can be categorized into two sectors, physical-based and physiological-based. Physical-based signals include facial expressions (Huang et al., 2019), body gestures (Reed et al., 2020), audio (Singh et al., 2019), etc. All these signals are easy to collect and show good emotion recognition performance. However, the dependability of the physical-based data can be equivocal as it can be easy for people to intentionally alter their reactions, which results in a false reflection of their real emotions. Physiological-based signals on the other hand include electroencephalogram (EEG), electrocardiogram (ECG), electromyogram (EMG), electrodermal activity (EDA)/galvanic skin response (GSR), blood volume pulse (BVP), temperature, photoplethysmography (PPG), respiration (RSP), and so on (Chen et al., 2021). While people may know the reasons why their signals are being collected, physiological signals relating to emotional states are hard to manipulate because they are controlled by the autonomic nervous system (ANS) (Shu et al., 2018). Compared to physical-based methods, emotion recognition based on physiological signal is less sensitive to societal and cultural differences amongst users and are acquired in natural emotional states (Betella et al., 2014). Physiological signals also perform better in detecting sounds than on beeps as they primarily reflect emotional states (Stuldreher et al., 2020).

Emotion should be well defined and approached in a quantifiable manner. Psychologists model emotions in two ways: discrete and multi-dimensional continuous (Liu et al., 2018; Mano et al., 2019; Yao et al., 2020). Discrete emotion theory claims that there is a small number of core emotions. They are usually limited and can barely differentiate heterogeneous emotions and composite mental states. Multi-dimensional emotion model on the other hand finds correlation among different discrete emotions which correspond to a higher level of a particular emotion. Valence-Arousal (V-A) space model has been widely used in affective computing research as a type of multi-dimensional continuous emotion model. Valence stands for a negative to a positive level of emotion that ranges from unpleasant to pleasant feelings. Arousal stands for low to high level of emotion that ranges from drowsiness to intense human excitement. The combination of valence and arousal space model enables the creation of continuous emotion models that captures both moderate and complex emotions needed for building accurate emotion recognition systems (Zhang et al., 2020). This study adopts the V-A space model for final emotion state classification.

**Figure 1:**
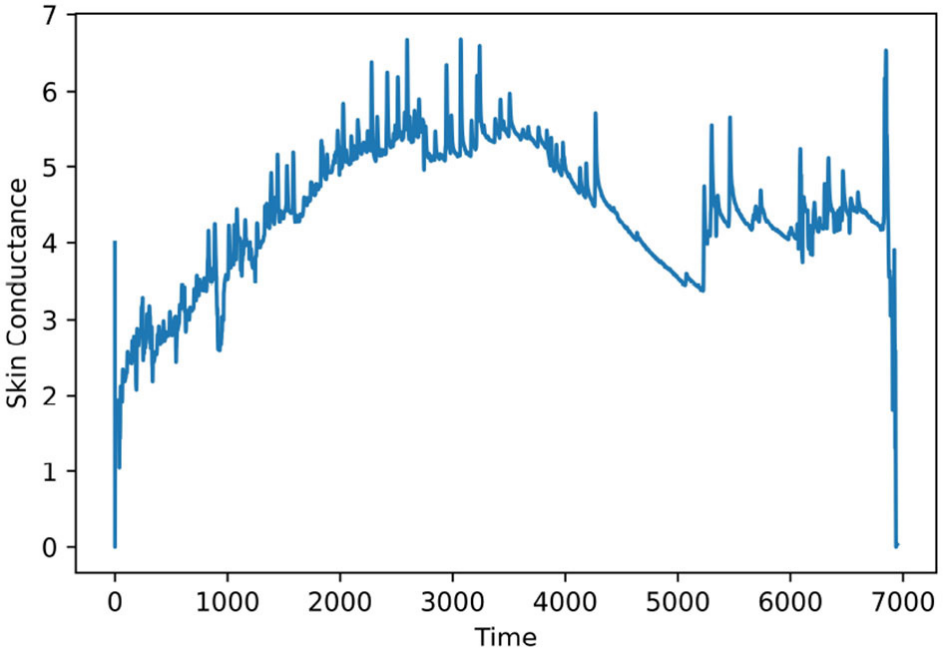
EDA signal of participant #39.

The electrodermal activity also known as galvanic skin response is a non-stationary signal. Figure 1 shows the EDA signal of participant #39 from the PAFEW dataset. Skin conductance response occurs when there is an induction of stimuli and may occur many times in short instances as shown in the figure. Portable low-cost EDA sensors (Milstein and Gordon, 2020) have been developed and applied in a physiological research context as signals become easier to collect. Prior works have connected electrodermal activities with biofeedback in diffusing brain activation as its cost effective and easy to apply treatment options are with no known risk (Gebauer et al., 2014). Researchers have begun combining and comparing the sufficiency of traditional machine learning and deep learning algorithms to predict users' mental states from EDA (Ayata et al., 2017).

Knowledge graph (KG) (Hogan et al., 2021) involves the acquisition and integration of information from data, creating relevant entities and relations, and predicting a meaningful output. As a new type of representation, KG has gained a lot of attention in the field of cybersecurity, natural language processing, recommendation systems, and human cognition (Jia et al., 2018; Guan et al., 2019; Chen et al., 2020; Qin et al., 2020; Yang et al., 2020). Wang (Wang et al., 2019) represented cognitive relations between different emotion types using a knowledge graph. Yu et al. (2020) studied a framework made up of non-contact intelligent systems, knowledge modeling, and reasoning to represent heart rate (HR) and facial features to predict emotional states. Farashi and Khosrowabadi (2020) used the knowledge of minimum spanning tree (MST) graph features derived from computational methods to classify emotional states. Wenbo et al. used knowledge embedding in a deep relational network to capture and learn relationships between cartoon, sketch, and caricature face recognition (Zheng et al., 2020). Gender and age are important factors that affect human emotion. To the best of our knowledge, there is no existing work on emotion recognition that attempts to combine gender and age with EDA/GSR statistical features (SF) to accurately model emotional states. In this paper, we propose an effective knowledge embedding graph model based on observed participants' gender and age to capture the relations between given entities. This has been lacking in EDA-Based Emotion Recognition Systems where researchers only focus on time, frequency, and time-frequency statistical features (SFs). We further propose a sophisticated feature fusion technique that exploits the knowledge embedding vectors as a weight to SFs. We then utilized a deep neural network to capture relevant complex information from participants' age and gender and predict emotional states.

The main contributions of this article is summarized as follows:

1) We propose an effective knowledge embedding graph model based on observed participants' gender and age to capture the relations between given entities.

2) We propose a sophisticated feature fusion technique that exploits the knowledge embedding vectors as a weight to SFs.

3) The proposed model shows better recognition accuracy when compared to other methods.

**Figure 2:**
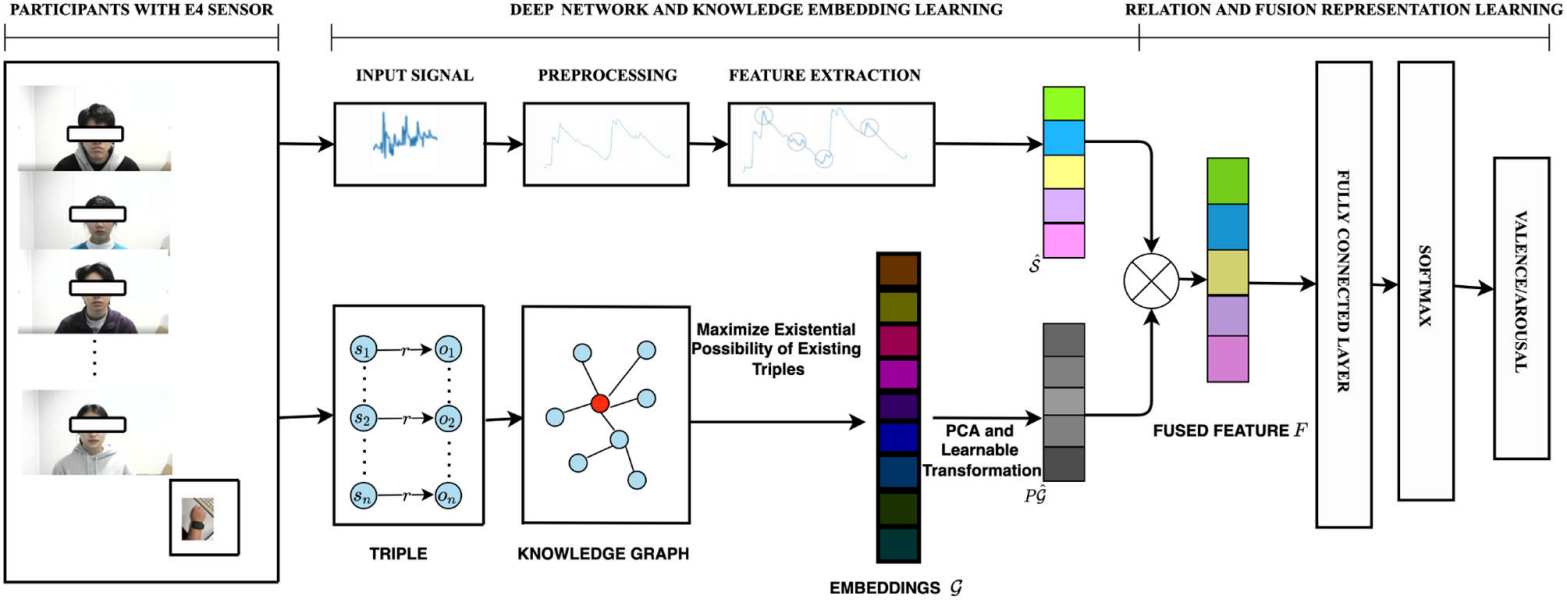
Overall implementation of the statistical features (SFs) and the observed knowledge graph features in accordance with the EDA signal. First, the network is used to extract EDA time and frequency SFs, S. We then construct a Gender-Age Relation graph (GARG) in a form of triples to learn relations and get embedding feature vectors. A transformation matrix *P* is introduced to exploit the graph embeddings. The fused feature *F* is gotten by combining the SFs and knowledge embeddings $\hat{G}$ after dimension reduction for training, validation, and testing. Finally, a fully connected neural layer and SOFTMAX is used for emotion state classification within the valence-arousal scale.

The overall implementation framework of this paper is illustrated in Figure 2.

The rest of the paper is organized as follows: Section 2 presents related background and approaches to EDA signals and knowledge graph and also presents our novel methodology and algorithms. Section 3 reports the experimental results. Section 4 discusses the novel approach and compares them with previous works. Finally, Section 5 concludes the study.

## 2. Materials and Methods

### 2.1. Related Background

Electrodermal activity-based emotion recognition has become popular in affective computing and cognitive developmental studies. Knowledge graphs have also been applied to embed entities in attempts to get more information from available data. Both ideas are frequently used nowadays in a variety of contexts and researchers have attempted to design frameworks to accurately solve today's problems.

### 2.2. EDA-Based Emotion Recognition

Electrodermal activity is a cheaper, easily collected physiological signal that reflects internal reactions to yield exhilaration. Deep learning algorithms (Yang et al., 2022) have become a catalyst in emotion recognition to capture time, frequency, and time-frequency information in EDA signals. Multilayer Perceptron (MLP), Convolutional neural network (CNN), Deep Belief Networks (DBN), Attention-Long Short Term Memory (A-LSTM) are often used. These algorithms with processors in the suggestive connection between neurons and layers can learn to extract relevant features for a reference task. In the study by Hassan et al. (2019), signals from EDA, Zygomaticus Electromyography (zEMG), and Photoplethysmogram (PPG) are fused using DBN for in-depth feature extraction to gear up a feature fusion vector. The vector is used to classify five discrete basic emotions, relaxed, happy, sad, disgust, and neutral. The study also used Fine Gaussian Support Vector Machine (FGSVM) together with radial basis kernel function to help classify the non-linearity in the human emotion classification. Song et al. also proposed an attention-long short term memory (A-LSTM) to extract discriminative features from EDA and other physiological signals to strengthen sequence effectiveness (Song et al., 2019). These studies did not use the gender and age information of participants as features to validate their results. Also, the performance of their approach did not quantify the contribution of the EDA signal but proves deep learning is effective in extracting emotional features.

### 2.3. Knowledge Graph

An effective way to learn representation from data is through embedding a KG into a vector space while maintaining its properties (Li et al., 2020). It involves knowledge triples *h, r, t* compiled of two entities *h* and *t* and a relation *r*. Face recognition studies have tried to study representational gaps using deep learning techniques to derive useful facial information (Cui et al., 2020). KG embedding can also be used to find vector representation of known graph entities by regarding them as a translation of entities in space. Human knowledge allows for a formal understanding of the world (Ji et al., 2022). For cognition and human level intelligence, knowledge graphs that represent structural relations between entities have become an increasingly popular research direction. The performance of these approaches shows that when KG is integrated, model performance is enhanced. Graph convolutional network has been studied for drug prediction in computational medicine (Nguyen et al., 2022). KG has been used for image processing and facial recognition but has never been used with physiological signals for emotion recognition. We attempt to address this issue.

### 2.4. Datasets

#### 2.4.1. The PAFEW Dataset

**Figure 3:**
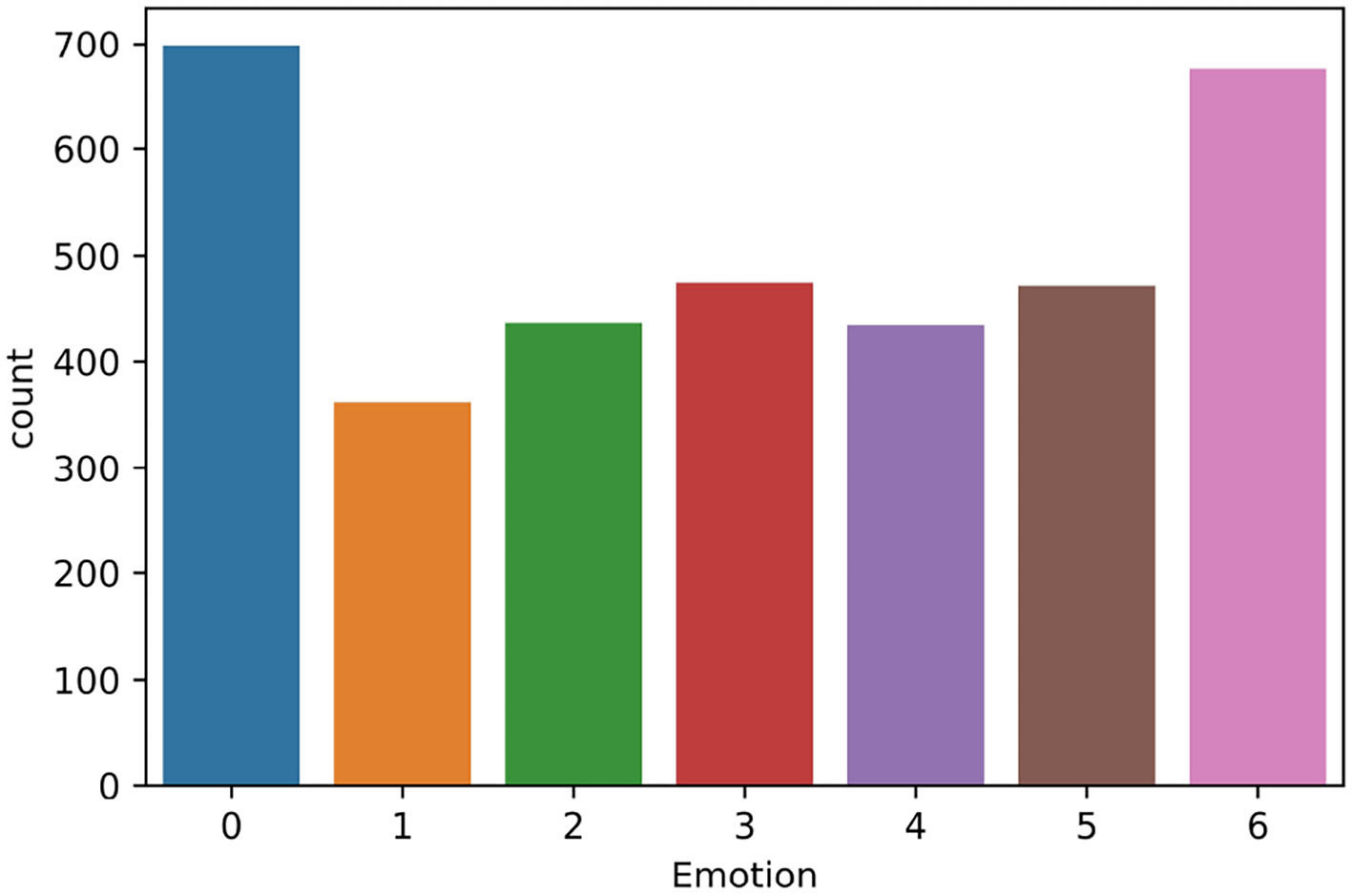
Imbalanced emotion class distribution in the PAFEW dataset.

**Figure 4:**
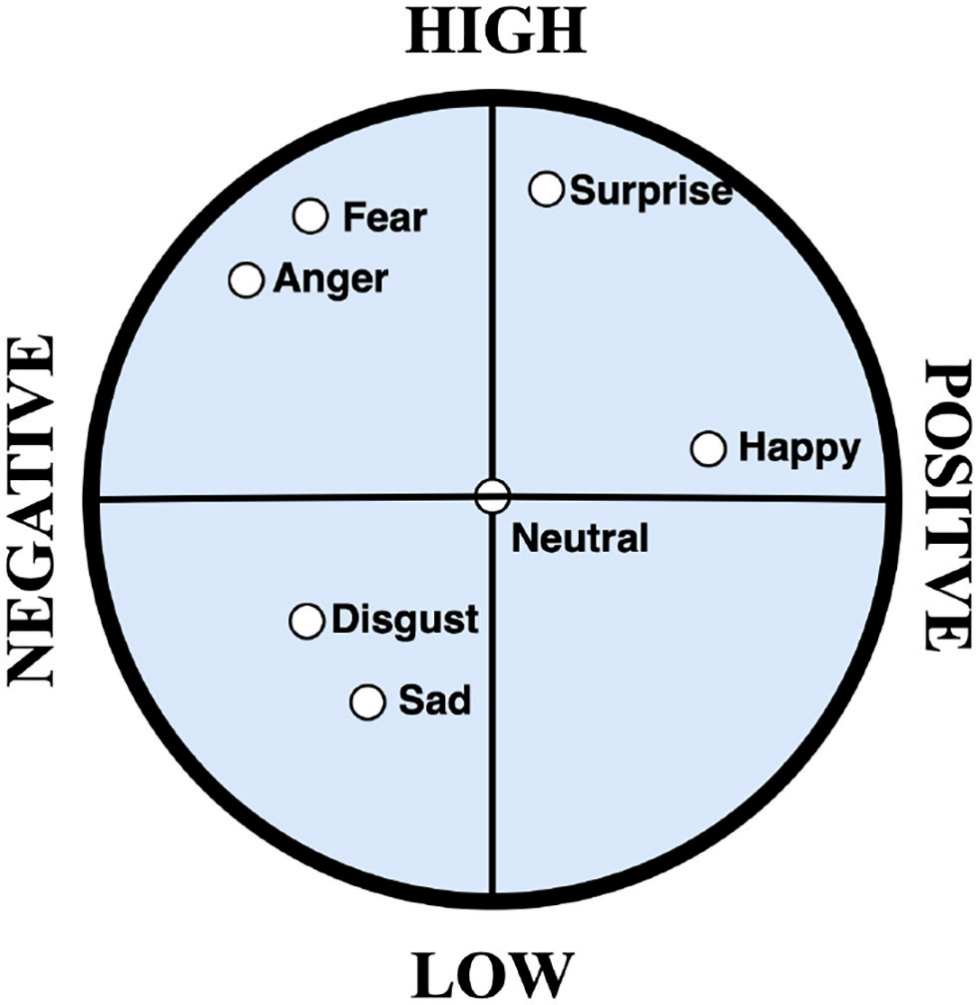
Valence-Arousal 2D Model: This is put into two separate groups. The first group is high arousal and low arousal. High arousal contains surprise, fear, anger, and happy. Low arousal contains disgust, neutral, and sad. Second group is positive valence and negative valence. Positive valence contains neutral, surprise, and happy. Negative valence contains fear, anger, sad, and disgust (Liu et al., 2020).

For the PAFEW dataset (Liu et al., 2020), there are 57 healthy students that participated in the experiment and each view about 80–90 video clips in all 7 emotion categories. Participants are aged between 18 and 39 years and were subjectively rated to keep them focused. The dataset is summarized in Table 1. Participants watched videos with one label continuously. This encourages them to be fully dissolved into one emotion before moving on to watching another video with a different label. The initial dataset was collected in a multi-class fashion (shown in Figure 3). From the figure, we can conclude that the PAFEW dataset is highly overlapped resulting in class imbalance. To tackle the imbalance issue, we subset target labels into the valence and arousal dimensions (shown in Figure 4). This is to more effectively capture both moderate and complex emotions. For example, fear and anger are two separate emotions which may differ from person to person but fall under high arousal and negative valence. Also, to be able to assess the performance metrics, we defined the total number of samples per emotion used to train and test the performance of the proposed method (seen in Table 2). Overall, we have a total of 3,554 data points.

**Table 1:** Summary of the PAFEW dataset.

**Attributes**		**PAFEW**
Length of sequence		480–6,040 ms
No. of sequence		4023
No. of participants		57
Max. participants per clip		9
Min. participants per clip		2
Mean participants per clip		5.2
	Happy	699
	Surprise	362
	Disgust	436
No. of sequence per expression	Anger	475
	Fear	434
	Sad	472
	Neutral	676

**Table 2:** List of independent data points for valence arousal classification.

**Valence**				**Arousal**			
**Positive**		**Negative**		**High**		**Low**	
Happy	699	Disgust	436	Happy	699	Disgust	436
Surprise	362	Anger	475	Fear	434	Sad	472
Neutral	676	Fear	434	Anger	475	Neutral	676
-	-	Sad	472	Surprise	362	-	-
Total	1737	Total	1817	Total	1970	Total	1584

#### 2.4.2. DEAP Dataset

The DEAP dataset (Koelstra et al., 2012) is made up of 32 participants that watched 40 video clips while their physiological signals and facial expressions are recorded. Participants are aged between 19 and 37 (mean age 26.9). Participants' rating was also collected. We used the GSR data/channel to design an emotion recognition system. A summary of the DEAP dataset is shown in Table 3.

**Table 3:** Summary of the DEAP dataset.

**Array name**	**Array shape**	**Array contents**
data	40 ×1 ×8,064	trial × channel × data
labels	40 ×2	trials × label

### 2.5. Methodology

In this section, we first illustrate the Gender-Age Relation Graph (GARG) and SF structures that are used in conducting the experiment. We also describe the weighted fusion representation learning which is the keystone of our solution.

#### 2.5.1. GARG Feature Learning Structure

Knowledge bases represent data by a directed graph with labeled edges (relations) between nodes (entities). We attempt to solve the problem of valence-arousal emotion recognition. Usually, the directed graph is represented by triples in the form (subject, predicate, and object). In our case, we use the gender and age information of a participant to construct the graph, so we have triples like (*Participant, AgeIs*, 28) and (*Participant, GenderIs, F*/*M*).

Formally speaking, we define our knowledge graph G=E×R×E:

- E: a set of entities;
- R: a set of relations.

Let $E=\left(e_{1},e_{2},...,e_{N_{e}}\right)$ represent the set of entities in the knowledge graph and let $R=\left(r_{1},r_{2},...,r_{N_{r}}\right)$ represent the set of all relation types. We represent each triple as *x*_*a, b, c*_ = (*e*_*a*_, *r*_*c*_, *e*_*b*_) and model its presence with a binary random variable *y*_*a, b, c*_∈{0, 1} indicating whether a triple exists or not. In this paper, we extract latent embedding features by maximizing the existential possibility of existing triples.

Following (Trouillon et al., 2016), we use complex embedding features. The complex embeddings of the subject, object, and their relation are denoted by *e*_*s*_, *e*_*o*_, and $w_{r}\in {\mathbb{C}}^{k}$, respectively. The scoring function of a triple is defined by:


(1) $$\begin{array}{l} f\left(s,o,r;\Theta \right)=Re\left(<w_{r},e_{s},{ē}_{o}>\right)\\ \;=Re\left(<\overset{K}{\underset{k=1}{\sum }}w_{rk},e_{sk},{ē}_{ok}>\right)\\ \;=\langle Re\left(w_{r}\right),Re\left(e_{s}\right),Re\left(e_{o}\right)\rangle \\ \;+\langle Re\left(w_{r}\right),Im\left(e_{s}\right),Im\left(e_{o}\right)\rangle \\ \;+\langle Im\left(w_{r}\right),Re\left(e_{s}\right),Im\left(e_{o}\right)\rangle \\ \;-\langle Im\left(w_{r}\right),Im\left(e_{s}\right),Re\left(e_{o}\right)\rangle , \end{array}$$

where *Re*(·) and *Im*(·), respectively, extract the real and imaginary parts of a complex vector, and Θ denotes the parameters corresponding to the model.

It is expected to score the correct triples (*e*_1_, *r, e*_2_) higher than incorrect triples $\left(e^{\prime },r,e_{2}\right)$ and $\left(e^{\prime },r,e_{1}\right)$ which is not equal to correct triples by one entity. Following Toutanova and Chen (2015), the conditional probability *p*(*e*_2_∣*e*_1_, *r*) for the object entity given the relation and the subject entity is defined as:


(2) $$\begin{array}{l} p\left(e_{2}|e_{1},r_{c};\Theta \right)=\frac{e^{f\left(x_{e_{1},e_{2},r_{c}}:\Theta \right)}}{\sum_{e_{2}^{\prime }\in Neg\left(e_{1},r_{c},?\right)}e^{f\left(x_{e_{1},e_{2},r_{c}}:\Theta \right)}} \end{array}$$

where (*e*_1_, *r*, ?) is a set of triples that do not match the object position in the relation triple. Since the entity number of such a set is large, the negative triples are randomly sampled from the full set. Similarly, given the relation and object, the conditional probability of the subject is defined as:


(3) $$\begin{array}{l} p\left(e_{1}|r_{c},e_{2};\Theta \right)=\frac{e^{f\left(x_{e_{1},e_{2},c}:\Theta \right)}}{\sum_{e_{1}^{\prime }\in Neg\left(?,r_{c},e_{2}\right)}e^{f\left(x_{e_{1}^{\prime },e_{2},c}:\Theta \right)}} \end{array}$$

Given the definition of conditional probability, we can maximize the probability of existing triples. Our training loss function is defined as the sum of the negative log-probabilities of observed triples with *L*2 penalty. Suppose *X* denotes the set of observed triples and λ is the trade-off parameter, the training loss is given by:


(4) $$\begin{array}{l} L\left(X,\Theta ,\lambda \right)=-\underset{x_{e_{1},e_{2},r_{c}}\in X}{\sum }log\;p\left(e_{2}|e_{1},r_{c}:\Theta \right)\\ \;-\underset{x_{e_{1},e_{2},r_{c}}\in X_{train}}{\sum }log\;p\left(e_{1}|r_{c},e_{2}:\Theta \right)\\ \;+\lambda \Theta^{T}\Theta . \end{array}$$

The algorithm of graph feature extraction is summarized in Algorithm 1.

**Algorithm 1:** GARG.

**Input:** Triples G={(E×R×E)}.
**Output:** Embedding Feature Vector.
**Procedure:**
1: Create entities (*s and o*) that will form a graph.
2: Initialize *x*_*a,b,c*_ based on relationship between entities and ensure its presence using *y*_*a,b,c*_∈{0, 1}.
3: **loop**
4: **for** each ϕ _*abc*_ **do**
5: Define the score triple using Equation (1).
6: Corrupt to generate Neg object triples $G^{-}=\left(E\times R\times E^{\prime }\right)$ at training for positive, or true triple.
7: Evaluate by defining filters to ensure no negative statements generated by the corruption procedure are actually positives by concatenating train and text sets.
8: **end for**
9: Update entity and relation embedding w.r.t gradients of Equation (4).
10: **end loop**

#### 2.5.2. SF Learning Structure

#### 2.5.3. Preprocessing

In this paper, we used PAFEW and DEAP databases for analysis. E4 wristband is used to collect EDA signals at a rate of 4 Hz in the PAFEW dataset. Participants were asked to watch videos of the same emotional label consecutively. We treat each emotion category as continuous in time for normalization. Data obtained varies significantly from 0 to 6.68 among participants. Min-max normalization is applied to reduce in-between participant differences. The 11-point median filter is used to remove noise, smoothen signal sequence. For the DEAP dataset, we used the preprocessed data. We also normalized the data to reduce intra- and inter-individual differences associated with emotional responses. The data is downsampled to 128 Hz. After, it is segmented into 60 s trials and 3 s pre-trials, baseline is removed.

#### 2.5.4. Feature Extraction

We extract time and frequency signals which is listed in Table 4. Features were extracted for the entire duration as participants watch emotional videos. For the PAFEW data, extracted features were categorized into three groups: basic statistical variables, first-order differential variables, and second-order differential variables. For the DEAP dataset, we used the toolbox for emotional feature extraction from physiological signals (TEAP) (Soleymani et al., 2017) to extract SFs. These features are in line with the work of Shukla et al. (2019) that extensively studied and reviewed EDA features across statistical domains relevant for emotion recognition. Our choice of features is easy to compute online, thus, making them advantageous in the future for real-time recognition.

**Table 4:** Features used in this research.

**Dataset**	**Features**	**Parameters**	**Discription**
		MeanEDA	Mean of signal
		KurtEDA	Kurtosis of signal
		SkewEDA	Skewness of signal
	Basic statistical features	MaxEDA	Maximum of signal
PAFEW		MinEDA	Minimum of Signal
		StdEDA	Standard deviation of signal
		VarEDA	Variance of Signal
	First order differential features	MeanAbsDiff	Mean of first order
		MeanNegativeDiff	Negative mean of first order differential
	Second order differential features	MeanSecAbsDiff	Mean of first order
		MeanSecNegativeDiff	Negative mean of second order differential
	Graph embedded features	Gender	Gender of participant
		Age	Age of participant
		No. of Peaks	Number of peaks in resistance
		Amplitude of Peaks	GSR peak amplitude
	Statistical Features	Rise time	Time taken to reach peak
DEAP		Statistical moments	Mean and Standard deviation
		Local minima	No. of local minima in GSR signal
	Graph Embedded Features	Gender	Gender of participant
		Age	Age of participant

#### 2.5.5. Graph Feature Weighted Fusion Representation Learning

We present our proposed weighted feature fusion mechanism for representation learning which effectively improves the performance of our model.

After training with the loss function (4), we extract the real part of the complex feature vector of the subjects as a graph embedding feature, and the feature matrix of all entities is denoted by G. High-dimensional space is required to embed the entities so that they are adequately distinguished, but the dimension is incompatible with the SF. Here, we use principal component analysis (PCA) to reduce the dimension to be the same as SF. The feature matrix after dimension reduction is denoted by $\hat{G}$.

To fuse SF S and graph embedding feature $\hat{G}$, one naive method is concatenation. Since they are extracted based on a totally different viewpoint, simple concatenation may not fuse them well. Here, we use a more sophisticated method by exploiting the graph embedding feature as the weight of the SF. Because the dimension order of the graph embedding feature does not naturally match the dimension order of SF, we introduce a learnable transformation matrix *P*. The fused feature is given by:


(5) $$\begin{array}{l} F=S⊗\left(P\hat{G}\right), \end{array}$$

where ⊗ denotes element-wise product. By taking *F* as the input of a neural network, we can obtain the transformation matrix *P* through training. Here, we use an MLP with the architecture of three hidden layers and an output layer. The dimension for the number of the hidden units is 64, 128, and 64. The output of the network is 2-dimension vector. We use the mean square error (MSE) criterion and Adam optimizer. Dropout is set at 0.2, batch-normalization and ReLU are applied after each hidden layer after which Softmax is employed for final classification. The whole procedure of our feature fusion learning method is summarized in Table Algorithm 2.

**Algorithm 2:** Weighted SF-GARG.

**Input:** Graph embedding feature $\hat{G}$ and statistical feature S
**Procedure:**
1: Extract embedding feature using **Algorithm 1** in high dimension and denoted as G
2: Use PCA to reduce dimension of feature matrix then denote as $\hat{G}$
3: Extract statistical feature and denote as S
4: Introduce a learnable feature transformation matrix *P*
5: Compute the fused feature *F* using Equation (5)
6: Define the neural network architecture
7: Take the fused feature *F* as input and train the network
**Output:** *P, W*

## 3. Results

We reported experimental results separately for each experiment to have a clearer assessment of our knowledge graph EDA-based approach.

Tables 5, 6 present the average results of classification recall, precision, accuracy, and F1-scores on PAFEW and DEAP. We also report the standard deviation of accuracy.

**Table 5:** Classification results (%) on the PAFEW dataset.

		**Valence**				**Arousal**			
**Features**	**Model**	**Recall**	**Precision**	**Accuracy**	**F1-Score**	**Recall**	**Precision**	**Accuracy**	**F1-Score**
	SVM	0.623	0.623	0.634 ±0.064	0.623	0.714	0.733	0.614 ±0.059	0.723
	KNN	0.752	0.752	0.763 ±0.057	0.752	0.713	0.713	0.690 ± 0.060	0.713
SF	Random forest	0.754	0.739	0.754 ± 0.058	0.746	0.724	0.741	0.724 ± 0.058	0.732
	Naive bayes	0.612	0.663	0.612 ± 0.063	0.636	0.691	0.691	0.654 ± 0.061	0.691
	MLP	**0.812**	**0.812**	**0.794**±0.052	**0.812**	**0.753**	**0.753**	**0.744**±0.057	**0.753**
SF-GARG	SVM	0.741	0.741	0.652 ± 0.058	0.741	0.734	0.724	0.633 ± 0.059	0.729
	KNN	0.789	0.789	0.745 ±0.054	0.789	0.745	0.745	0.745 ±0.058	0.745
	Random forest	0.778	0.778	0.778 ± 0.055	0.778	0.756	0.767	0.745 ± 0.056	0.761
	Naive bayes	0.717	0.732	0.690 ± 0.059	0.724	0.724	0.704	0.678 ±0.060	0.714
	MLP	**0.847**	**0.862**	**0.828**±0.046	**0.854**	**0.815**	**0.793**	**0.770**±0.054	**0.804**

*The bold values are the highest accuracy obtained*.

**Table 6:** Classification results (%) on the DEAP dataset.

		**Valence**				**Arousal**			
**Features**	**Model**	**Recall**	**Precision**	**Accuracy**	**F1-Score**	**Recall**	**Precision**	**Accuracy**	**F1-Score**
SF	SVM	0.690	0.700	0.590 ± 0.081	0.695	0.705	0.732	0.690 ± 0.078	0.718
	KNN	0.750	0.690	0.700 ± 0.082	0.719	0.767	0.745	0.734 ±0.077	0.756
	Random forest	0.754	0.702	0.733 ± 0.081	0.727	0.752	0.752	0.713 ± 0.076	0.752
	Naive bayes	0.614	0.589	0.623 ± 0.087	0.601	0.754	0.744	0.691 ± 0.077	0.749
	MLP	**0.774**	**0.774**	**0.723**±0.074	**0.774**	**0.789**	**0.783**	**0.761**±0.729	**0.786**
SF-GARG	SVM	0.745	0.749	0.770 ±0.077	0.747	0.700	0.720	0.690 ± 0.079	0.710
	KNN	0.740	0.733	0.714 ±0.078	0.736	0.745	0.767	0.734 ± 0.075	0.756
	Random forest	0.740	0.740	0.720 ±0.078	0.740	0.750	0.756	0.714 ± 0.076	0.753
	Naive bayes	0.720	0.710	0.680 ±0.080	0.715	0.740	0.740	0.689 ±0.078	0.740
	MLP	**0.805**	**0.802**	**0.793** ±0.070	**0.803**	**0.804**	**0.801**	**0.802**±0.071	**0.798**

*The bold values are the highest accuracy obtained*.

The results are based on leave-one-out training and testing, i.e., training all but one subject data, and using that one subjects data for testing. The results for SF features are consistent with previous studies. However, the GARG feature embeddings give a boost in model performance, hence improvement in classification results. This is because our graph embedding feature vectors can effectively learn the representation between participants' gender and age and their respective emotional labels. Also, because the GARG features are extracted differently and require a high dimensional space to effectively mine related information. Furthermore, directly combining them with the SF features will be inappropriate. We, therefore, use PCA to match their dimensional space to the SFs. Hereby, we used them as a weight to the statistical features to enhance model performance. The tables also clearly show that, a weighted combination of SF-GARG features yield better performance than those of the SF features alone. MLP clearly shows the highest performance in all tables compared to SVM, KNN, RF, and NB.

The Nemenyi test (Demšar, 2006) and Friedman test (Friedman, 1937) are used to show statistical significance of our method. To detect the differences between multiple methods across multiple test results, the Friedman test, which is a non-parametric statistical test, is used. Its null hypothesis states that - all methods have the same performance. The Nemenyi test is used to distinguish whether the performances of the methods are significantly different if the null-hypothesis is rejected. In the PAFEW dataset, on the arousal scale, we calculated *F*_(5,)_ = 3.57 for accuracy and *F*_(5,)_ = 3.75 for F1 score. On the valence scale, we calculated *F*_(5,)_ = 3.69 for accuracy and *F*_(5,)_ = 3.89 for F1 score. This showed *p* < 0.05. Similarly, in the DEAP dataset on the arousal scale, we calculated *F*_(5,)_ = 3.63 for accuracy and *F*_(5,)_ = 3.76 for F1 score. On the valence scale, we calculated *F*_(5,)_ = 3.68 for accuracy and *F*_(5,)_ = 3.74 for F1 score which showed *p* < 0.05. The critical distance (CD) of the Nemenyi test is defined as follows: $CD=q_{\alpha }\sqrt{\frac{k\left(k+1\right)}{6N}}$, where *q*_α_ is a default critical value of 0.05, *k* denotes the number of methods (*k* = 5) in this work, and *N* the number of result groups. For PAFEW, *N* = 57 and for DEAP, *N* = 32. This resulted in no overlaps with methods suggesting that our proposed methods are statistically different in performance with compared methods.

**Figure 5:**
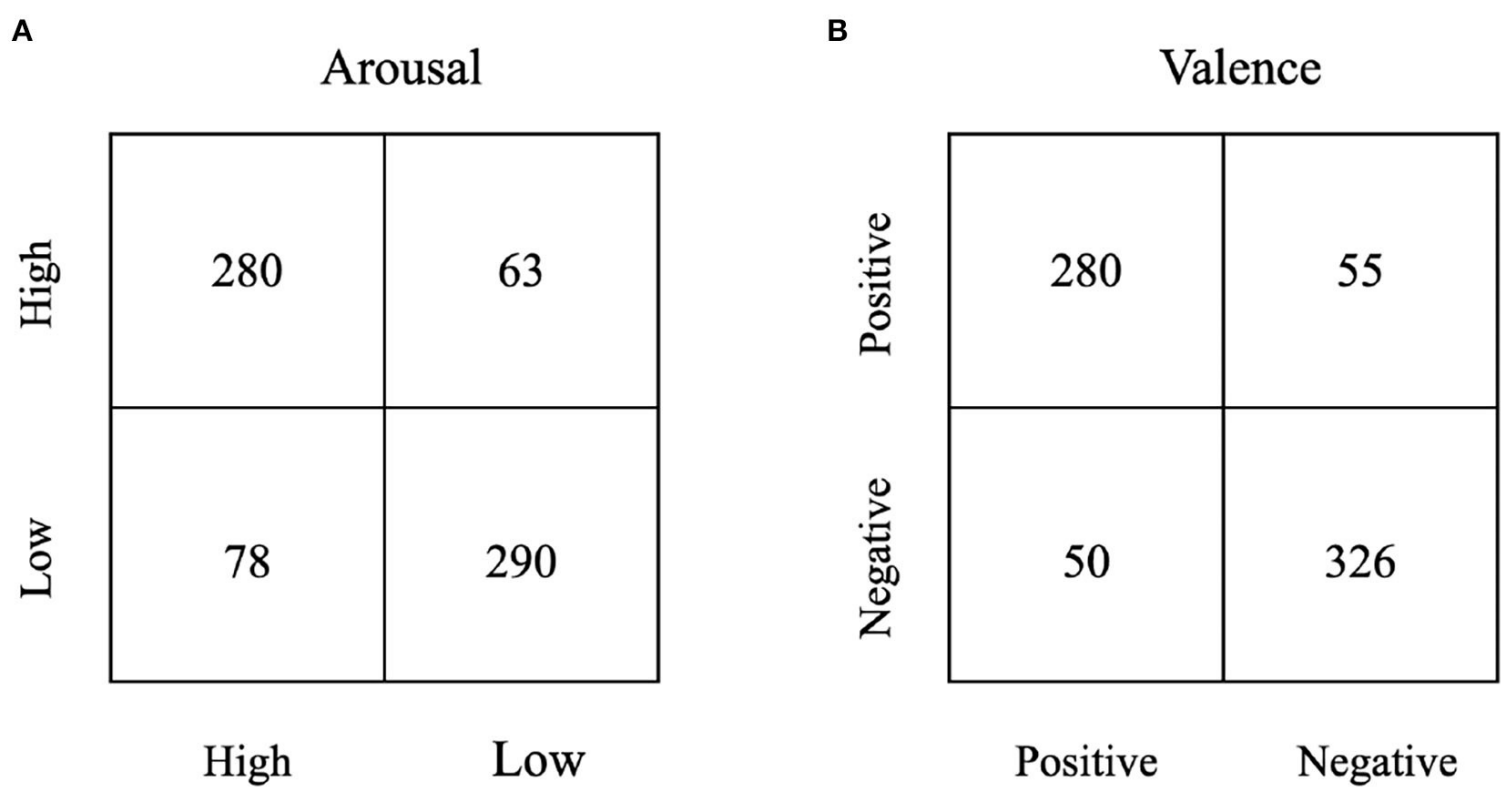
Sample confusion matrix of the classification accuracy on the PAFEW dataset, **(A)** is for Arousal Scale, **(B)** is for Valence Scale.

Figure 5 is the confusion matrix for the classification results on the PAFEW dataset and shows a clearer picture of our model performance.

**Figure 6:**
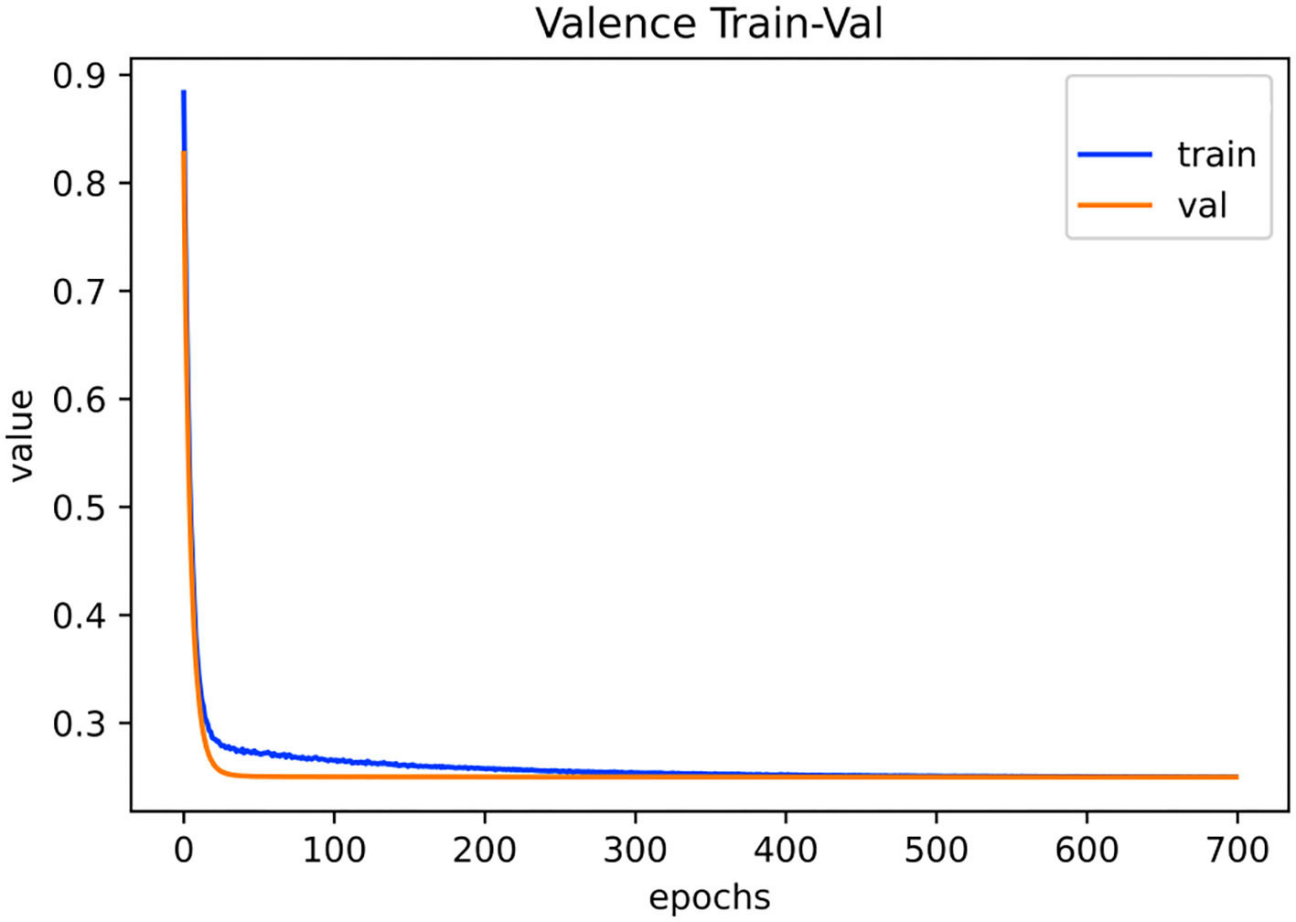
Visualization of training and validation loss on PAFEW dataset during the valence classification. Configuration consists of five layers trained for 700 epochs with a learning rate of 0.001.

**Figure 7:**
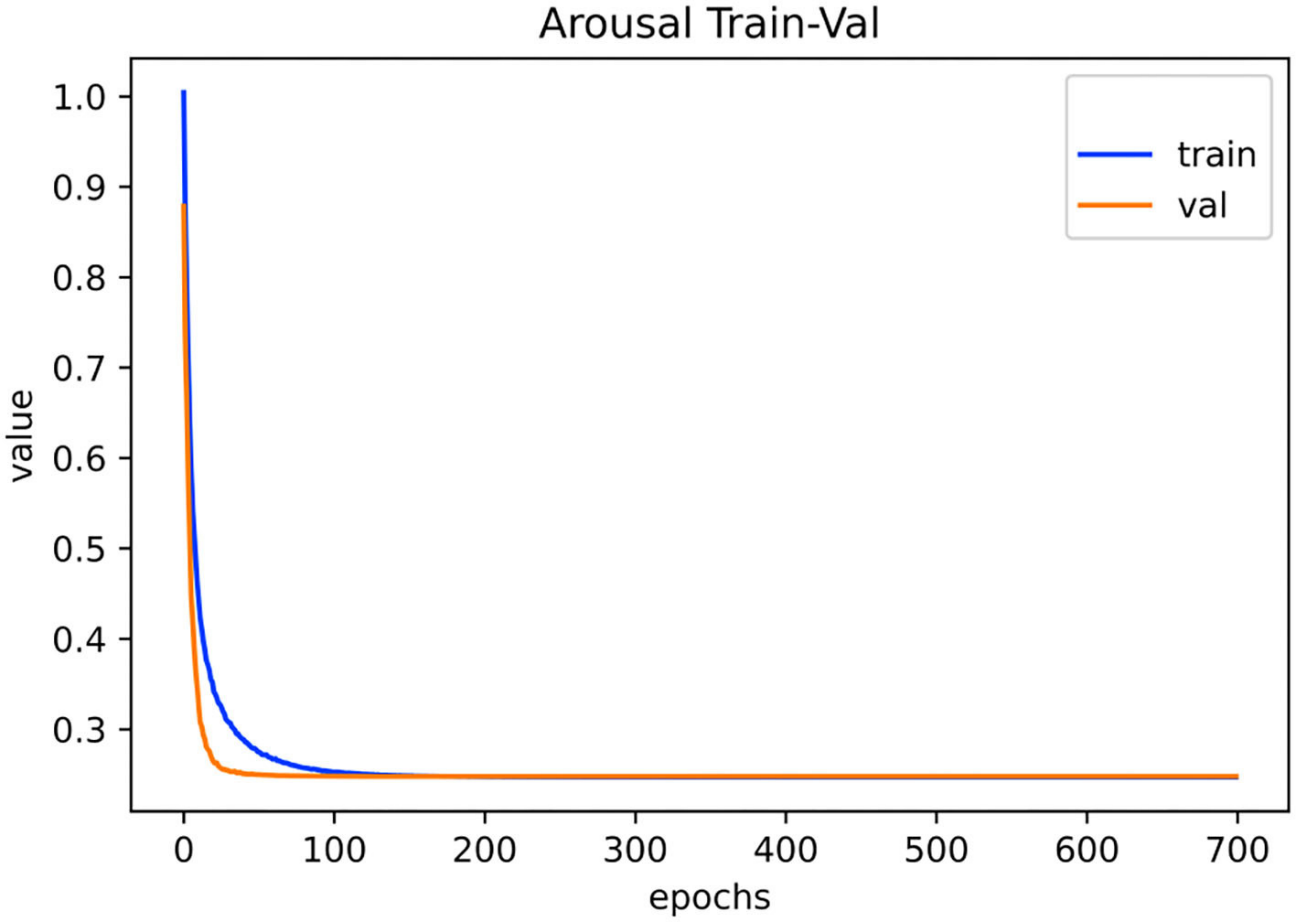
Visualization of training and validation loss on PAFEW dataset during the arousal classification. Configuration consists of five layers trained for 700 epochs with a learning rate of 0.001.

Figures 6, 7 show the training and validation loss on the PAFEW dataset for both valence and arousal classification. Our method reduces the complexity of the model with respect to the reduction of dimension of the complex embeddings and the number of layers in our neural network model. In the training phase, the time taken to train the model is relatively fast. The figures show that our model is able to reduce variance of bias and finds real relationships between graph and SF vectors from the EDA signal and their emotional labels.

## 4. Discussion

In this paper, we focused on two kinds of issues. 1) Knowledge graph generation using participant's gender and age information and 2) Weighted feature fusion issue for EDA emotion classification system. In generating a knowledge graph using gender and age, we created triple entities and drew relationships between EDA signals, participants' gender and age, and their respective emotion labels. The weighted feature fusion issue is an algorithmic idea that exhibits the performance of the proposed method in an effective way for EDA emotion applications. We also deliberated the advantages of MLP over other machine learning methods.

Regarding knowledge graph generation, results indicated that our deep learning approach can capture relevant complex information from participants' gender and age and predict emotional states correctly. On the contrary, SVM, KNN, RF, and NB could not fully mine such complex information but averaged consistent results higher than only SF features which also proves GARG features as effective for learning representation in EDA. The deep learning approach can also capture invisible features that traditional methods cannot. The results also show that our model can effectively identify true positive results (recall) than previous EDA-based approach.

Pertaining to the weighted feature fusion issue, the proposed sophisticated algorithm outruns existing methods. Unlike previous methods where features from other signals are directly concatenated, we introduced a transformation matrix that exploits the gender-age graph embedding features as a weight to the SF and performed element-wise multiplication. Table 7 compares our work with previous studies. Our model is able to reach an F1-measure of 85.4% and 80.4% for valence and arousal, respectively, for PAFEW dataset. On the DEAP dataset, our model reaches an F1-measure of 80.3% on the valence scale and 79.8% on the arousal scale. In comparison with Koelstra et al. (2012) and Soleymani et al. (2017) that uses machine learning algorithms, the features extracted did not particularly reflect participants' affective states. Our deep learning approach is data driven and based on training data. Also, the GARG embedded features reflect participants' affective state in the valence-arousal scale. Hence, the robustness of our method compared to theirs. Additionally, Ganapathy et al. (2020), Zhang et al. (2020), and Liu et al. (2020) uses DL but our model was manifestly superior with regard to training speed with low SD in both valence and arousal dimension. Table 8 summarizes all compared methods with respect to some qualitative features. It is easily observed that our proposed approach possesses two kinds of qualities—integrating knowledge graph embedding vectors and EDA features and excellent complexity order those other methods do not.

**Table 7:** Accuracy and F1-Score comparison with state-of-the-art-research using electrodermal activity (EDA) signals.

				**Valence score**		**Arousal score**	
**Paper**	**Dataset**	**Input signals**	**Val**	**Accuracy**	**F1-Score**	**Accuracy**	**F1-Score**
Ganapathy et al., 2020	DEAP	EDA	Leave-one-out	0.721	0.713	0.754	0.791
Our Work	DEAP	EDA, graph embedding	Leave-one-out	**0.793**	**0.803**	**0802**	**0.798**
Our Work	PAFEW	EDA, graph embedding	Leave-one-out	**0828**	**0.854**	**0.770**	**0.804**

*The bold values are the highest accuracy obtained*.

**Table 8:** Taxonomy of the compared approaches to EDA-Based emotion recognition.

**Method**	**Knowledge graph embedding**	**Deep learning**	**Wearable devices**
Ganapathy et al., 2020	No	Yes	No
SF-GARG	Yes	Yes	Yes

The proposed knowledge graph and EDA-based method take advantage of a single EDA module and graph embedding tricks to improve fusion performance. Our approach does not consume more computation time compared with other methods that use multiple kernel combination techniques. The introduction of the learnable transformation matrix enables the feature space to form an implicit combination. This is less computationally expensive compared to other methods. The multilayer neural network employed exhibits strength and flexibility in representation learning hence superior performance regarding fusion capabilities.

Finally, none of the previous works utilized participants' GARG embeddings as weight to statistical features to optimize their approach. This is an important advantage of our proposed approach. Investigating GARG embeddings and accurately combining them with SFs lead to a 4% and 5% increase in performance on the PAFEW dataset and 3% and 2% on the DEAP dataset for valence and arousal, respectively.

In the future, we can try to explore more physiological signals like EEG, EMG, and BVP with knowledge graph embeddings and devise new fusion techniques to improve emotion classification results.

## 5. Conclusion

This paper investigates the feasibility of employing GARG embeddings features and EDA/GSR Statistical Features (SF) to build an emotional state classification system. We proposed an effective knowledge graph method that uses gender and age information that is mostly ignored in feature analysis to predict participants' emotional states. We then extract SFs from the EDA/GSR data consistent with cognitive research. Finally, we introduced a weighted fusion strategy that uses GARG embeddings as a weight to SFs to improve classification performance. We evaluated our study on PAFEW and DEAP datasets. The results obtained show the superiority of our approach when compared to previous works.

## Data Availability Statement

Publicly available datasets were analyzed in this study. The data can be found here: https://sites.google.com/view/emotiw2020 and https://www.eecs.qmul.ac.uk/mmv/datasets/deap/.

## Author Contributions

HP, XXi, KG, and XXu proposed the idea. HP conducted the experiment, analyzed the results, and wrote the manuscript. XXi was in charge of technical supervision and provided revision suggestions. KG analyzed the results, reviewed the article, and was in charge of technical supervision. XXu was in charge of technical supervision and funding. All authors contributed to the article and approved the submitted version.

## Funding

This work was supported in part by the National Natural Science Foundation of China under Grant U1801262, Guangdong Provincial Key Laboratory of Human Digital Twin (2022B1212010004), in part by the Key-Area Research and Development Program of Guangdong Province, China, under Grant 2019B010154003, in part by Natural Science Foundation of Guangdong Province, China, under Grant 2020A1515010781, and Grant 2019B010154003, in part by the Guangzhou key Laboratory of Body Data Science, under Grant 201605030011, in part by Science and Technology Project of Zhongshan, under Grant 2019AG024, in part by the Fundamental Research Funds for Central Universities, SCUT, under Grant 2019PY21, Grant 2019MS028, and Grant XYMS202006, and in part by Guangdong Philosophy and Social Sciences Planning Project, under Grant GD20CYS33.

## Conflict of Interest

The authors declare that the research was conducted in the absence of any commercial or financial relationships that could be construed as a potential conflict of interest.

## Publisher's Note

All claims expressed in this article are solely those of the authors and do not necessarily represent those of their affiliated organizations, or those of the publisher, the editors and the reviewers. Any product that may be evaluated in this article, or claim that may be made by its manufacturer, is not guaranteed or endorsed by the publisher.
